# The prevention of anxiety in children through school-based interventions: study protocol for a 24-month follow-up of the PACES project

**DOI:** 10.1186/1745-6215-15-77

**Published:** 2014-03-13

**Authors:** Paul Stallard, Gordon Taylor, Rob Anderson, Harry Daniels, Neil Simpson, Rhiannon Phillips, Elena Skryabina

**Affiliations:** 1 Department for Health, University of Bath, 22-23 Eastwood, Bath BA2 7AY, UK; 2 University of Exeter Medical School, The Veysey Building, Salmon Pool Lane, Exeter EX2 4SG, UK; 3 Department of Education, University of Oxford, 15 Norham Gardens, Oxford OX2 6PY, UK; 4 Sirona Care and Health, Headquarters building, Bath BA2 5RP, UK; 5 Wales School for Primary Care Research, Cardiff University School of Medicine, Heath Park, Cardiff CF14 4XN, UK

**Keywords:** Prevention, Anxiety, Schools, Children

## Abstract

**Background:**

Anxiety in children is common and incapacitating and increases the risk of mental health disorders in adulthood. Although effective interventions are available, few children are identified and referred for specialist treatment. Alternative approaches in which prevention programmes are delivered in school appear promising. However, comparatively little is known about the best intervention leader (health care–led vs. school-led), long-term effects or the primary preventive value of such programmes.

**Methods/Design:**

Preventing Anxiety in Children through Education in Schools, or PACES, is a pragmatic cluster randomised controlled trial evaluating the effectiveness of a cognitive-behavioural therapy prevention programme (FRIENDS) on symptoms of anxiety and low mood in 9- to 10-year-old children. Forty-one schools were randomly assigned to one of three conditions: school-led FRIENDS, health care–led FRIENDS or treatment as usual. Assessments were undertaken at baseline, 6 months and 12 months, with the primary outcome measure being the Revised Child Anxiety and Depression Scale score at 12 months. Secondary outcome measures are changes in self-esteem, worries, bullying and life satisfaction.

**Discussion:**

This protocol summarises the procedure for the 24-month follow-up of this cohort. The study will determine the medium-term effectiveness of an anxiety prevention programme delivered in schools.

**Trial registration:**

ISRCTN23563048

## Background

Anxiety and depressive disorders in children are common. The investigators in the American Great Smoky Mountains Study found that, during a 3-month period, 2.4% of children ages 9 to 16 years fulfilled the diagnostic criteria for an anxiety disorder and 2.2% met the criteria for a depressive disorder [1]. Similar rates were found in the British Mental Health Survey, in which 3.7% of 5- to 15-year-olds had a current anxiety disorder and 1% had a depressive disorder [2]. Comorbidity of anxiety and depression is common [3,4], with cumulative rates suggesting that, by 16 to 17 years of age, 15% to 18% of children will have experienced an impairing emotional disorder of anxiety or depression [1,4].

Longitudinal studies highlight that child mental health disorders persist into adulthood. In the Dunedin birth cohort study, approximately 52% to 55% of young adults with depression or anxiety met the diagnostic criteria for a mental health disorder before 15 years of age, with 75% receiving a first diagnosis before the age of 18 [5]. Childhood anxiety increases the risk of anxiety, depression, substance misuse and educational underachievement in early adulthood [6]. Similarly, childhood depression increases the risk of suicide, subsequent depression and substance misuse. The associated health-related burden and economic and societal costs are considerable, and the need to improve the mental health of children is being increasingly recognised as a priority at the global level [7-9].

Whilst effective psychological treatments are available, few children with emotional disorders receive them. Surveys undertaken in the United Kingdom and the United States have revealed that approximately one-third of children with anxiety disorders and less than one-half with depressive disorders had sought or received help from specialists over a 1- to 3-year period [10,11]. The persistence of emotional disorders, as well as the immediate and future burden, and the limited reach of treatment services have led to interest in alternative approaches to improve the mental health of children.

School-based mental illness prevention programmes offer an attractive alternative to traditional treatment approaches. Systematic reviews have highlighted that anxiety and depression prevention programmes can be effective, although the results have been widely variable [12-15]. Methodologically, many of these studies are poorly designed, sample sizes are small, comparisons with other active interventions are lacking and follow-up is limited. Implementation studies whereby efficacious interventions are evaluated in suitably powered trials under everyday conditions are comparatively few and have delivered disappointing results. Recent evaluations of large, well-designed depression prevention programmes delivered for children in schools, for example, have failed to find intervention effects [16-18]. Although the results of anxiety prevention programmes have tended to be more encouraging, recent implementation trials have failed to find positive effects [19,20]. Two important issues that will influence programme effectiveness in preventing mental health problems in children are the ways in which the programme is provided (universally versus targeted) and who delivers the intervention (health care professionals versus teachers) [12,14,15].

Prevention programmes can be provided universally (that is, to all of an identified population, regardless of risk status) or targeted toward those at risk of developing mental health disorders or showing early signs of a disorder [21]. Universal programmes avoid the need for costly screening, fit better within complex school timetables, are less stigmatising and provide opportunities for primary prevention. This last point is important because many trials of prevention programmes have focused on demonstrating evidence of treatment effects (that is, reducing current symptom levels) rather than on preventive effects, such as a reduction in the emergence of new cases [22]. Universal interventions tend to have a smaller effect than targeted programmes, however, and, in times of economic pressure, may not be considered the best use of limited resources [23,24].

In terms of delivery, health care professionals or graduates tend to be more effective than trained school staff in delivering depression prevention programmes [15]. Reviewers have found no difference in effectiveness between health care professionals and school staff in the delivery of anxiety prevention programmes [12]. Direct comparisons within prevention trials between health care professionals and school staff have seldom been undertaken, however, so the most effective form of prevention programme delivery is not known.

Of the emotional health prevention programmes that have been developed, FRIENDS for Life has been identified as one of the more efficacious programmes [13,25]. FRIENDS is based on cognitive-behavioural therapy and develops children’s skills to enhance emotional regulation, coping mechanisms and thinking styles. A pragmatic randomised controlled trial is currently underway in the United Kingdom to compare the effectiveness of universally delivered health care–led FRIENDS, school-led FRIENDS and usual school provision of personal, social and health education (PSHE) at 12 months after initiation [26]. The purpose of the Preventing Anxiety in Children through Education in Schools (PACES) trial is to assess the medium-term (24 months) effects. First, differences in emotional health between health care– and school-led FRIENDS and usual school provision of PSHE at 24 months will be investigated. Second, the effects of the three conditions at 24 months on children with high and low levels of anxiety at baseline will be explored.

## Methods/Design

PACES is a pragmatic, three-arm, parallel cluster randomised controlled trial, with school used as the unit of allocation and individual participants being the unit of analysis [26]. Participating children were recruited during school year 5 (ages 9 to 10 years), and all were eligible unless they were not attending school (for example, due to long-term sickness or excluded from school) or did not participate in PSHE lessons for religious or other reasons. Parent consent and child assent were obtained before completing the assessments.

### Power calculation

The study is powered to detect a difference between the FRIENDS programme (health care–led and school-led) and usual school provision of PSHE. On the basis of an intracluster correlation coefficient of 0.02, 28 pupils per class, 90% consent and 80% retention, effect sizes in the range of 0.28 to 0.30 standard deviations are detectable with 80% power and 5% two-sided α and including 45 to 54 schools (that is, 1,134 to 1,360 consenting pupils). On the basis of these assumptions, a cohort of 907 to 1,088 pupils at 12 months was required.

### Randomisation

After recruitment, schools in South West England were randomised on a 1:1:1 ratio to health care–led FRIENDS, school-led FRIENDS or usual school provision of PSHE. Trial arms were balanced for school size, number of students and classes, number of mixed classes, level of educational attainment and preferred time-tabling. A statistician with no other involvement in the study randomly selected one sequence from a subset with the most desirable balance properties.

### Interventions

Interventions were delivered in the academic year spanning September 2011 to July 2012. The FRIENDS interventions consisted of nine 60-minute weekly sessions delivered to whole classes of children (that is, universal delivery). Children had their own workbooks, and group leaders had a detailed session plan specifying key learning points, objectives and core activities for each session.

For health care–led FRIENDS, each session was led by two trained facilitators working alongside the class teacher. All facilitators had at least an undergraduate university degree in a relevant discipline, an appropriate professional background and/or experience in working with children and young people. Initial two-day training and ongoing fortnightly supervision were provided by accredited FRIENDS trainers.

In school-led FRIENDS, sessions were led by a trained teacher or a member of the school staff and were supported by two facilitators. School staff attended the same two-day training session and were offered ongoing supervision.

For usual school provision of PSHE, children participated in the usual PSHE sessions provided by the school. All schools were following a UK national programme designed to develop self-awareness, management of feelings, motivation, empathy and social skills [27]. The sessions were planned and provided solely by the teacher and did not involve any external input from the research team.

### Twenty-four-month assessment

#### Recruitment strategy

The PACES cohort transitioned to secondary school in September 2013. This cohort will be invited to participate in the 24-month assessment according to our recruitment strategy drawing upon suggestions from systematic reviews [28].

First, opt-in reply slips and a project information sheet with the university and PACES logos will be sent from schools to parents of all children who participate in the PACES study. Second, playground recruitment visits will be undertaken at the end of the day, when parents collect their children. These recruitment steps will allow informal discussions about the project and an opportunity for parents to allow their children to participate in the study. Third, a range of PACES publicity materials, including the project contact details (for example, pencils, refrigerator magnets, stress balls and message bugs), will be handed out to children during these visits. Fourth, a PACES Facebook page will be established as a way of allowing parents to contact the study team and opt to have their children participate in the study. Fifth, a £30 financial incentive will be offered to compensate parents and children for their time in completing the assessments.

## Consent and ethical approval

Signed parent consent and child assent are required for participation in the 24-month assessment. The 24-month assessment was approved by the University of Bath Research Ethics Approval Committee for Health.

### Outcome measures: child report

Assessments were undertaken in the original PACES project at baseline and at 6 and 12 months by self-completed questionnaires administered by researchers blinded to allocation arm. The 24-month assessment will involve the same measures that have been completed on the three previous occasions.

The primary outcome for our long-term follow-up is the Revised Child Anxiety and Depression 30-item Scale (RCADS-30) score assessed at 24 months [29]. This self-report questionnaire is used to assess anxiety and depression symptoms that correspond to diagnostic criteria published in the *Diagnostic and Statistical Manual of Mental Disorders, Fourth Edition* (the DSM-IV). The 30-item scale will be used, which comprises 6 subscales to assess social phobia, separation anxiety, obsessive-compulsive disorder, panic disorder, generalised anxiety disorder and major depressive disorder. Items are rated on a four-point Likert scale. The RCADS-30 has good internal consistency, test–retest stability and convergent and divergent validity [30,31].

Child-reported secondary outcomes will be used to assess self-worth and acceptance, worry, bullying, life satisfaction and school concerns at 24 months. The Rosenberg Self-Esteem Scale [32] is a ten-item, self-completed questionnaire related to overall feelings of self-worth or self-acceptance. The items are answered on a four-point scale ranging from strongly agree to strongly disagree. The Rosenberg Self-Esteem Scale has demonstrated good reliability and validity across a large number of different sample groups, including children aged 7 to 12 years, and is one of the most commonly used and best-known measurement tools for self-esteem.

The Penn State Worry Questionnaire for Children is a self-report questionnaire that measures the tendency of children to engage in excessive, generalised and uncontrollable worry [33]. Items are rated on a four-point Likert scale to assess how strongly each item applies to the child. The original scale consists of 14 items. The 11-item version used in this trial has improved psychometric properties when used with children aged 8 to 12 years [34].

The Olweus Bully/Victim Questionnaire is the most widely used questionnaire to assess the nature and extent of bullying amongst schoolchildren. The two global items assessing the frequency of self-reported bullying and being the victim of bullying will be used.

Subjective well-being and satisfaction with six aspects of life (school, appearance, family, home, friendships and health) and overall life satisfaction are assessed using a seven-point scale. These aspects were selected from among the 12 domains identified as contributing to the subjective well-being of children [35].

The School Concerns Questionnaire is a 20-item scale assessing worries about starting secondary school [36]. Items cover organisational concerns (that is, changing classes, remembering equipment), social concerns (that is, making new friends, being bullied) and academic concerns (for example, homework, being able to do the work). Each item is rated on a ten-point scale assessing the extent of worry.

### Outcome measures: parent report

Parents will first complete the same measures they completed at baseline and at 6 and 12 months. The Strength and Difficulties Questionnaire is a brief, widely used behavioural screening questionnaire for 3- to 16-year-olds completed by parents and teachers. It asks about 25 attributes, some positive and others negative. These 25 items cover emotional symptoms, conduct problems, hyperactivity and/or inattention, peer relationship problems and prosocial behaviours, which, added together, generate a total difficulties score [37,38].

Second, parents will complete the parent version of the Revised Child Anxiety and Depression 30-item Scale (RCADS-30-P). This scale covers the same 30 items completed by the children. The RCADS-P has high internal consistency, good test–retest reliability and good convergent and divergent validity [39].

### Outcome measures: economic

All children will complete the self-report Child Health Utility–9D (CHU-9D) at 24 months [40]. The CHU-9D has been developed specifically for children ages 7 to 11 years and is a validated measure covering nine different domains of health-related quality of life.

A subgroup of parents (*n* = 307) was interviewed at baseline and at 6 months (*n* = 284) using the Client Receipt of Service Questionnaire to assess health, social care and educational service use [41]. Parents in this subgroup who opt to participate in the 24-month assessment will be asked to complete this questionnaire again, along with the assessment of life events and a screen of parent physical and mental health that they previously completed.

### Statistical analysis

Data analysis will be undertaken by study members blinded to allocation arm. Analysis and presentation of data will be in accordance with the Consolidated Standards of Reporting Trials (CONSORT) guidelines, in particular with the extension to cluster randomised trials [42]. The primary comparative analyses will be conducted on an intention-to-treat (ITT) basis with due emphasis placed on confidence intervals for the between-arm comparisons.

We will use descriptive statistics to assess balance between trial arms at baseline. The primary outcome will be assessed by ITT without imputation. To take appropriate account of the hierarchical nature of the data, we will use multivariable mixed-effects regression to compare mean RCADS-30 scores at 24 months for health care–led FRIENDS versus school-led FRIENDS versus usual school provision of PSHE, with adjustments made for baseline RCADS-30 scores and randomisation variables. These analyses will be repeated for secondary outcomes. We will undertake a secondary analysis using interaction terms in the regression model to explore differences at 24 months between randomised arms and the baseline variable (RCADS scores of 0 to 38 (low anxiety) and 39 and higher (high anxiety)).

## Discussion

Adequately powered randomised trials assessing the effectiveness of anxiety prevention programmes, when implemented under everyday conditions, are required before the widespread use of these programmes can be advocated. Our study will compare an efficacious programme (FRIENDS) delivered by health care professionals or school staff against usual school provision of PSHE. The study will assess the effect of intervention leaders and the impact of the programme on both high- and low-symptom children to determine both reduction in symptomatology and whether the intervention has a preventive effect on low-symptom children. The 24-month assessment will allow the medium-term effects to be determined.

### Study status

Forty-one schools consented to participate in PACES and were randomised, and one withdrew before baseline assessments were undertaken. The remaining 40 schools had 1,448 eligible participants of whom 1,362 (94%) consented to participate in the study. Twelve-month outcome data were obtained from 1,257 consenting children (94%), resulting in a larger cohort than initially predicted.

Recruitment for the 24-month assessment started in June 2013. Assessments started in October 2013 and are scheduled to be completed by May 2014.

## Abbreviations

CHU-9D: Child Health Utility Index; CONSORT: Consolidated Standards of Reporting Trials; ITT: Intention to treat; PACES: Preventing Anxiety in Children through Education in Schools; RCADS: Revised Child Anxiety and Depression Scale.

## Competing interests

The authors declare that they have no competing interests.

## Authors’ contributions

PS, GT RA, NS, HD and RP conceived the study and led the bid to secure funding for this work. They have contributed to the development of the protocol and are involved in managing and advising on the project. ES is the trial manager and has contributed to the development of the protocol and the drafting of this paper. All authors read and approved the final manuscript.
